# Assessment of knee flexor muscles strength in patients with patellar instability and its clinical implications for the non-surgical treatment of patients after first patellar dislocation - pilot study

**DOI:** 10.1186/s12891-021-04636-4

**Published:** 2021-08-28

**Authors:** Krzysztof Małecki, Jarosław Fabiś, Paweł Flont, Anna Fabiś-Strobin, Kryspin Niedzielski

**Affiliations:** 1 Clinic of Orthopaedic and Traumatology, Polish Mother’s Memorial Hospital Research Institute, Rzgowska 281/289, 93-338 Lodz, Poland; 2 Department of Arthroscopy, Minimally Invasive Surgery and Sports Traumatology, Medical University of Lodz, Żeromskiego 113, 90-549 Lodz, Poland

**Keywords:** patella dislocation, Hamstring strength, Isokinetic assessment, Children

## Abstract

**Background:**

Biomechanical studies indicate that during outward rotation of the tibia and the valgus knee joint position, the patella is shifted in the lateral direction. After first-time patellar dislocation, the dynamic position of the femur in relation to the tibia plays an important role in joint stability, because the medial stabilizer of the patella (mostly the MPFL) is damaged or inefficient. The most important factor in controlling the rotational movement of the tibia in relation to the thigh are the hamstring muscles. The aim of the study therefore is to determine whether patients with patellar instability have a significant weakness in the knee flexor muscles, which can predispose to recurrent dislocations. This is an important consideration when planning the rehabilitation of patients with first-time patellar dislocation.

**Methods:**

The study enrolled 33 patients with confirmed recurrent patellar dislocation, including six patients with bilateral involvement. In the study group, the hamstring muscles (both sides) were evaluated at velocities of 60 and 180 deg/s for the following parameters: peak torque, torque at 30 degrees of knee flexion, angle of peak torque and peak torque hamstring to quadriceps ratio (H/Q ratio).

**Results:**

In the recurrent patellar dislocation group, a statistically significant weakness in knee flexors was observed for both angular velocities compared to age and gender normative data. No such relationship was observed in the control group of heathy subjects. In patients with one-sided dislocation, no differences were found in knee flexors peak torque, torque at 30 degrees of knee flexion, angle of peak torque or H/Q ratio between the healthy and affected limbs for either angular velocity.

**Conclusions:**

In patients with recurrent patellar dislocation, knee flexors strength is decreased significantly in both the unaffected and affected limbs. This may indicate a constitutional weakening of these muscles which can predispose to recurrent dislocations.

**Trial registration:**

The study was retrospectively registered on ClinicalTrials.gov (NCT04838158), date of registration; 22/03/2021.

## Introduction

Although patients with recurrent, i.e. at least second, dislocation of the patella are qualified for surgical treatment, there is a lack of clear agreement on how to treat those with the first-time dislocation [1–4]. The routine management the first dislocation is conservative treatment and rehabilitation; however, there are no unequivocal guidelines and no data indicating that such treatment prevents recurrence [5–9]. Recurrent dislocations occur in 15 to 72% of cases of first-time dislocation, depending on the risk factors [10]. The most frequently-mentioned risk factors include patella alta, increased TT-TG distance, trochlear dysplasia, patellar tilt, bilateral occurrence of the dislocation, skeletal immaturity, genus valgus, increased femoral anteversion and familial occurrence [8, 9, 11]. To our knowledge, no analysis of the significance of muscle strength as a risk factor for recurrence of the dislocation has been conducted. While the need to restore the strength of the quadriceps muscle is widely accepted, the role of hamstring strength has been underestimated [12–16].

The dynamic position of the femur in relation to the tibia is influenced by the flexors and extensors of the knee joint. Biomechanical studies indicate that, in a closed kinematic chain, the outward rotation of the tibia and the valgus knee joint position shifts the patella in a lateral direction. Patellar dislocation leads to an injury or stretching of the medial patellofemoral ligament (MPFL); this occurs when the patella moves out of the intercondylar groove, typically during the last 20–30 degrees of knee joint extension [17]. The dynamic position of the femur in relation to the tibia in patients after first-time patellar dislocation plays a key role in its stability, because the medial stabilizer of the patella (mostly the MPFL) is damaged or inefficient. As the hamstring muscles are mainly responsible for controlling the rotational movement of the tibia in relation to the thigh, the present study will examine their strength in patients with recurrent patellar dislocation.

The aim of the work is to confirm whether patients with patellar instability have a statistically significant weakness in the knee flexor muscles that can increase the probability of recurrent dislocation. Such knowledge could be of value when performing the rehabilitation of patients with first-time patellar dislocation.

## Materials and methods

The study enrolled 33 patients with confirmed recurrent patellar dislocation, including six patients with bilateral involvement. The study group consisted of 20 girls and 13 boys with a mean age of 16.2 years at the time of the study (range: eight to 17 years, SD 2.7). For the purpose of the study, a control group of 18 healthy subjects (12 girls and six boys) mean 15.7 years (range:13 to 17 years, SD 1.5), was also recruited. A three-month standard rehabilitation programme with thigh muscle strengthening was performed after the last recurrent dislocation, followed by an individual home exercise programme. Isokinetic testing was performed six months after the incident. Surgical patellar stabilisation was performed in all cases after testing.

The following inclusion criteria were applied: recurrent patellar dislocation, correctly-completed testing protocol, age under 18 years at the time of testing. Subjects with osteochondral fracture and those with a history of knee surgery were excluded.

Each patient underwent an isokinetic examination on the Biodex Multi-Joint System - Pro (Biodex Medical Systems, Inc. New York, USA). In the study group, hamstring muscles (both sides) were evaluated for peak torque (PT), torque in 30 degree of the knee flexion (T 30 deg.), angle of peak torque (APT) and peak torque hamstring to quadriceps ratio (H/Q ratio) at velocities of 60 and 180 deg/s. The lower speed (60 deg/s) was used to reflect the maximum isokinetic resistance used in everyday situations, and the knee can be subjected to extreme loads during sports; therefore, this speed was used to highlight any deficits in the maximum load and to indicate a potential need for correction.

Torque values in 180 deg/s are well-standardized and commonly-used isokinetic criteria for admission to training after knee injuries and surgery. When measuring PT, a standard parameter used for the isokinetic evaluation of the knees, the knee joint function is also evaluated in terms of its stability. APT is known to change in cases of patellar instability, and its normalization is a reliable indicator of full recovery of knee function in patellar instability, especially when the value is compared with the healthy knee. The H/Q ratio plays a key role in allowing a return to full activity, and in planning an effective correction procedure that ensures an appropriate hamstring/quadriceps balance; in addition, in the case of unilateral changes, the healthy knee can be used as a point of reference to allow for precise correction in the case of existing deficits. Another important parameter is torque at 30 degrees of knee flexion (T 30 deg.), which indicates the torque value at the position where the patella is the most likely to be malpositioned during active knee motion.

Isokinetic measurements were performed by a trained, experienced physiotherapist. The patients were instructed on methodology of testing. After a warm-up, the patients performed 10 full repetitions of measurements, with the best result being recorded. None of the patients reported pain, apprehension or instability during the isokinetic testing; however most demonstrated a typical positive apprehension test during passive lateralisation of the patella. In cases of unilateral dislocation, no patellar laxity was evident, nor were any apprehension tests positive on the healthy side. The peak torque (PT) of both the test and control limbs were then compared with the normative data for age and sex available in the literature [18, 19]. The obtained PT values for the control group of healthy subjects were also compared with normative data. In addition, the PT, T 30 deg., APT and H/Q ratio values for involved and uninvolved knee flexor strength were compared in the study group. Depending on the data distribution, the nonparametric Wilcoxon’s test or the parametric t-test for the related data was applied. Statistical significance was assumed for *p* < 0.05. The study was approved by Polish Mother Memorial Hospital Review Board and registered on ClinicalTrials.gov (NCT04838158). Informed consent was obtained and the rights of subjects were protected.

## Results

In the recurrent patellar dislocation group, a statistically significant weakness in knee flexors peak torque was observed for both angular velocities compared with age- and sex-related normative data (*p* < 0.001) (Tables 1, 2). In the control group of healthy subjects, no such weakness was observed for either angular velocity (*p* = 0.67, *p* = 0.29) compared to normative data (Tables 1, 2). In 27 patients with one-sided dislocation, hamstring peak torque of healthy limbs was significantly decreased compared to normative data (*p* < 0.001, *p* = 0.004). However, in these 27 patients with one-sided dislocation, no difference was observed between the healthy and affected limbs with regard to PT, T 30 deg., APT or H/Q ratio for either angular velocity (Table 3).

**Table 1:** Results of peak torque at 60 deg/s (z-value; Wilcoxon’s test)

Peak Torque 60	X (mean)	z-value	*p*-value
Normative data (for age and sex)	72.2 Nm	z = −5.535	p < 0.001
knees with recurrent dislocation *n* = 39	39.7 Nm		
Normative data (for age and sex)	73.13 Nm	z = −4.681	p < 0.001
Uninvolved knees *n* = 27	53.01 Nm		
Normative data (for age and sex)	75.5 Nm	z = −1.826	*p* = 0.0672
Control group of healthy subjects *n* = 18	66.3 Nm

**Table 2:** Results of peak torque at 180 deg/s (z-value; Wilcoxon’s test)

Peak Torque 180	X (mean)	z-value	p-value
Normative data (for age and sex)	51.1 Nm	z = −2.594	*p* = 0.016
knees with recurrent dislocation n = 39	27.9 Nm		
Normative data (for age and sex)	51.9 Nm	z = −2.881	p = 0.004
Uninvolved limbs n = 27	30.6 Nm		
Normative data (for age and sex)	45.9 Nm	z = −1.048	*p* = 0.2937
Control group of healthy subjects n = 18	53.9 Nm

**Table 3:** Results of peak torque (PT), torque at 30 degrees of knee flexion (T 30 deg.), angle of peak torque (APT) and peak torque hamstring to quadriceps ratio (H/Q ratio) for 60 and 180 deg/s. (z-value; Wilcoxon’s test, t-value; parametric t-test)

		X (mean)	statistical value	p-value
PT 60	involved limbs n = 27	39.3 Nm	z = −0.7786	*p* = 0.436
	uninvolved limbs n = 27	45.1 Nm		
PT 180	involved limbs n = 27	29.0 Nm	z = −0.084	*p* = 0.9362
	uninvolved limbs n = 27	30.7 Nm		
H/Q ratio 60	involved limbs n = 27	56.7 Nm	z = −1.585	*p* = 0.114
	uninvolved limbs n = 27	44.9 Nm		
H/Q ratio 180	involved limbs n = 27	57.7 Nm	z = −0.574	*p* = 0.567
	uninvolved limbs n = 27	50.7 Nm		
T 30 deg. 60	involved limbs n = 27	25.3 Nm	z = −0.773	*p* = 0.441
	uninvolved limbs n = 27	28.2 Nm		
T 30 deg. 180	involved limbs n = 27	23.5 Nm	z = −1.63	*p* = 0.103
	uninvolved limbs n = 27	22.5 Nm		
APT 60	involved limbs n = 27	63 deg.	t = 1.182	*p* = 0.248
	uninvolved limbs n = 27	66 deg.		
APT 180	involved limbs n = 27	48 deg.	t = 1.11	*p* = 0.277
	uninvolved limbs n = 27	52 deg.		

## Discussion

Our findings indicate that patients with recurrent patellar dislocation demonstrate significantly decreased knee flexors strength in both unaffected and affected limbs in comparison with normative data. Creating a control group of healthy patients not affected with patellar instability confirmed the validity of the normative data: no differences in peak torque were found between the normative data and the control group.

Our findings also demonstrate that patients with unilateral recurrent patellar dislocation are also subject to bilateral flexors weakness. In such cases, bilateral hamstring muscles weakness may indicate a constitutional weakening of these muscles. In these patients, it is possible to improve the muscular strength of the flexors through intensive rehabilitation. Rehabilitation is especially important in patients after the first-time patellar dislocation when the passive medial knee stabilizers have been injured.

In the rehabilitation of patients with patellar instability, attention is paid only to the strength of the quadriceps muscle; however, the vastus medialis obliquus (VMO), a part of the quadriceps, generates a force vector moving the patella medially [12–16]. Despite this, there is no mention in the literature concerning the possibility of targeted strengthening of the medial hamstrings to counteract the rotational-valgus malalignment mechanism of patellar dislocation.

In cases of multidimensional anteroposterior knee instability with anterior cruciate ligament insufficiency, it is very important to strengthen the hamstrings, which act as active knee stabilizers [20–26]. A similar rotational mechanism has been observed in anteroposterior knee instability and patellar dislocation. The literature describes the coincidence of patellar dislocation with the MCL and ACL injury [27–29]. By enhancing the strength of the knee flexors, i.e. active knee stabilizers, it is possible to limit the number of torsional injuries that can lead to patellar dislocation, which is highly probable in the case of previous MPFL damage. This may suggest that rehabilitation is very important in the treatment of first-time patellar dislocation, and surgical treatment may not always be necessary; however, this is only a pilot study and such findings need further qualification. Even so, our results suggest that in patients with the first-time dislocation, it may be advisable to attempt to strengthen the knee flexors before making the decision to perform surgery.

To our knowledge, no previous study has precisely assessed knee flexor strength in patients with patellar dislocation without surgery; however, there is a noticeable trend towards extending the indications for surgery in the first dislocation of the patella. An analysis of flexor strength (peak torque) at speeds of 90 and 240 deg/s by Askenberger et al. did not reveal any significant weakening in comparison with the healthy limb, which was also confirmed in the present study [30]. It is important to note that the constitutional weakening of flexors in this group of patients may affect also the healthy limb, and a deficiency can also be observed compared to the control group. In the same publication, the authors provide further arguments for expanding research into the conservative treatment of first-time patellar dislocations. The results of surgical treatment (MPFL suturing) and conservative management (knee brace and rehabilitation program) in patients after first-time dislocation were compared during a two-year follow-up period: the authors observed recurrence rates of 22% after the surgery and 43% after conservative treatment. Furthermore, non-surgical treatment was associated with more favourable scores on subjective functional scales [30]. Similarly, better functional outcomes in patients after non-surgical treatment were presented by Zimmerer et al. [1].

As this was a pilot study and only a limited number of adolescent patients were available, no power analysis was performed. Our findings need to be supported by cohort studies of patients after first-time dislocation, who underwent strengthening of flexor muscles correlated with the rate of recurrences and isokinetic muscle assessment. Our findings suggest that, based on the published normative data for age and sex and the results obtained in the control group, the weakening of flexors demonstrated by patients might be constitutional, affecting also the healthy limbs, and is not an effect of patellar instability.

Our study could provide guidance to target rehabilitation in patients with first-time dislocation, who have no clear indications for surgery, such as concomitant osteochondral fracture. Isokinetic testing, a repetitive and objective examination, should be used to monitor the progress of rehabilitation, indicated by an increase in hamstrings strength. We propose that first time patella dislocation should be still treated conservatively, and emphasize that quadriceps strengthening exercises should be supplemented by intensive and dedicated knee flexors training in these patients in any case.

## Data Availability

Along with ClinicalTrials registration conditions we are ready to share the raw data upon reasonable request. Responsible person: Krzysztof Małecki e-mail: krzynormal@wp.pl.

## Abbreviations

MPFL: medial patellofemoral ligament
H/Q ratio: peak torque hamstring to quadriceps ratio
TT-TG distance: tibial tuberosity-trochlear groove distance
SD: standard deviation
PT: peak torque
T 30 deg: torque in 30 degree of the knee flexion
APT: angle of peak torque
VMO: vastus medialis obliquus
ACL: anterior cruciate ligament
MCL: medial collateral ligament
